# A phase 2 study of combined chemo-immunotherapy with cisplatin-pembrolizumab and radiation for unresectable vulvar squamous cell carcinoma

**DOI:** 10.1186/s12967-020-02523-5

**Published:** 2020-09-14

**Authors:** Oladapo Yeku, Andrea L. Russo, Hang Lee, David Spriggs

**Affiliations:** 1 Division of Hematology-Oncology, Department of Medicine, Harvard Medical School, Massachusetts General Hospital, Boston, USA; 2 Department of Radiation Oncology, Harvard Medical School, Massachusetts General Hospital, Boston, USA; 3 Biostatistics Center, Harvard Medical School, Massachusetts General Hospital, Boston, USA

**Keywords:** Vulvar cancer, Pembrolizumab, Chemoradiation, Immunotherapy, Chemoimmunotherapy

## Abstract

**Background:**

Unresectable or metastatic vulvar cancer has relatively poor outcomes despite chemotherapy-sensitized radiation therapy and combination cytotoxic therapy. Despite the virus-associated and immunogenic nature of this disease, novel immunotherapy options that exploit this advantage are currently lacking. Platinum agents such as cisplatin have been shown to prime dendritic cells for T-cell costimulation, promote downregulation of inhibitory checkpoint molecules, and sensitize tumor cells to cytotoxic T-cell killing. Radiation therapy has also been shown to promote immunogenetic cell death as monotherapy and in combination with cisplatin. In combination with pembrolizumab, cisplatin-sensitized radiation is hypothesized to increase overall response rates and recurrence-free survival in patients with vulvar cancer, via induction of an anti-tumor inflammatory response.

**Methods:**

We propose a single-arm phase II clinical trial of pembrolizumab combined with cisplatin-sensitized radiation therapy for women with unresectable, locally advanced, or metastatic vulvar cancer. The first three patients with locally advanced or unresectable disease will receive cycle 1 of pembrolizumab followed by a break and resumption of pembrolizumab at cycle 4 and as part of a safety cohort. All other patients, including the fourth patient with locally advanced/unresectable disease, will receive weekly cisplatin and pembrolizumab every 3 weeks, concurrently with daily radiation therapy. Following the completion of Cis-RT, patients will continue pembrolizumab maintenance for a total of 12 cycles. Archived tissue will be used for HPV status, MSI status, PD-L1, and TIL stratification post hoc. Imaging will be performed at baseline and every 3 cycles (21-day cycles) as per standard-of-care. Laboratory analysis will occur on the first day of each cycle.

**Discussion:**

The combination of cisplatin-sensitized radiation and immune checkpoint blockade has not been evaluated in the upfront setting for vulvar cancer. In this rare malignancy, there are limited interventional clinical trials. This trial is designed to be as accessible as possible by allowing patients to receive cisplatin and radiation locally according to accepted standard-of-care while receiving pembrolizumab and adverse event monitoring at a centralized site. A robust suite of translational correlative studies has also been built into the trial to evaluate tumor-directed immune activation.

*Trial registration* NCT04430699

## Background

Vulvar cancer represents 4% of gynecologic malignancies in the United States with an estimated incidence of 6190 women diagnosed in 2018. Of these, 20% are expected to die from disease [1]. The primary treatment for locally advanced or recurrent metastatic disease is highly-individualized, and typically involves some combination of surgery, radiation therapy (RT) with or without radiosensitization and platinum-based doublet chemotherapy [2]. Patients with locally advanced or metastatic disease at presentation have a 5-year survival of 53% and 19% respectively [3]. Patients with recurrent metastatic disease have survival rates between 14 and 15% [2]. Furthermore, no standard-of-care exists for patients who recur or relapse after primary therapy. Platinum-based combination chemotherapy for advanced or recurrent metastatic vulvar cancer has an overall response rate (ORR) of 40% [4], while single-agent paclitaxel has a response rate of 12% [5]. These have corresponded to median progression-free survival rates of 10 months [4] and 2.6 months [5] for combination and single-agent therapy respectively.

For patients with early stage disease, surgical resection with sentinel lymph node evaluation or bilateral inguinofemoral lymph node dissection is the standard. Determination of surgical margins and lymph node involvement informs the decision for re-excision or adjuvant external beam radiation (RT). Patients with lymph node involvement could also be considered for RT with or without concurrent chemotherapy with cisplatin, cisplatin plus fluorouracil (5-FU) or 5-FU plus mitomycin-C [6].

For patients with regional lymph node metastasis, extra nodal extension of the tumor, or fixed/ulcerated nodal metastasis (FIGO III/IVA), upfront surgical debulking results in significant morbidity and mortality [7, 8]. Single-agent platinum therapy is inefficacious for this disease, as illustrated by the absence of any clinical responses to cisplatin monotherapy [9]. Chemoradiation has been shown to offer significant improvements in response rate, relapse-free survival and overall survival over RT in patients with locally advanced disease [10]. As such, upfront chemoradiation has been the generally employed standard. GOG 205 evaluated the clinical response rates in patients with locally advanced squamous cell vulvar carcinoma treated with cisplatin-sensitized radiation therapy [11]. They reported a 64% response rate, allowing for many of these patients to subsequently undergo consolidative surgery. The addition of consolidative surgery after chemoradiation does not confer any significant increase in overall survival over chemoradiation alone [12]. Although the addition of chemotherapy to radiation has been shown to improve survival time to up to 44 months in patients with node-positive disease [13], there is room to increase recurrence free survival in this patient population.

### Rationale for immunotherapy and platinum-sensitized radiotherapy

Platinum agents such as cisplatin, carboplatin and oxaliplatin have long been hypothesized to promote immunogenic modulation outside of their DNA-platinum adduct formation and inhibition of DNA replication [14, 15]. Lesterhuis et al. showed that human dendritic cells exposed to cisplatin induced significantly increased T-cell proliferation [16]. Furthermore, T-cells activated by platinum-sensitized DC’s secreted increased levels of IFN-γ and IL-2 [16]. Importantly, the authors found that this effect was in-part due to down-regulation of PD-L1 and PD-L2 on DCs. Finally, they found that cisplatin-mediated STAT6 dephosphorylation led to PD-L1/2 downregulation [16]. In other reports, upregulation of mannose-6-phosphate (M6P) by cisplatin sensitized tumor cells to granzyme-B killing, and cytotoxic T-cell-medicated killing [17]. Cisplatin, has also been shown to induce immunogenic cell death via modulation of STAT signaling [18].

Ionizing radiation (RT) therapy induces IFN-γ, type I IFN production and PD-1/PD-L1 expression on tumor cells [19]. When combined with cisplatin, the immunogenic effect of RT is further potentiated via calreticulin exposure, release of ATP, induction of programmed death receptor 1-ligand (PD-L1) and high-mobility protein box-1 (HMGB-1) [20, 21]. Finally, there are several preclinical and clinical studies supporting the rational combination of immune checkpoint inhibitors with RT. For instance, combination therapy resulted in increased tumor infiltration of cytotoxic CD8 T-cells, decreased regulatory T-cells and myeloid derived suppressor cells in melanoma and glioma tumor models [22, 23]. Furthermore, combination therapy enhanced antigen-cross presentation via upregulation of major histocompatibility complex (MHC) class I (MHC-I) [24] potentially priming the immune system for checkpoint blockade.

Lastly, immunotherapy maintenance after immunogenic interventions such as local ablative therapy or radiation therapy has been shown to be beneficial. For instance, patients with non-small cell lung (NSCLC) cancer treated with maintenance pembrolizumab after ablative therapy on a phase 2 study demonstrated statistically significant improvement in progression free survival (PFS) compared to historic controls [25]. In another report by Bersanelli et al. [26], patients who revived concurrent immunotherapy with radiotherapy had a longer PFS compared to patients who received radiation alone. A randomized placebo-controlled phase 3 trial evaluated the role of maintenance durvalumab in patients with NSCLC who had received chemoradiotherapy and found significant improvements in overall response rate, 12-month PFS, 18-month PFS and duration of response [27]. Notably, these combinations were well-tolerated.

## Methods

### Aim and study design

**Fig. 1:**
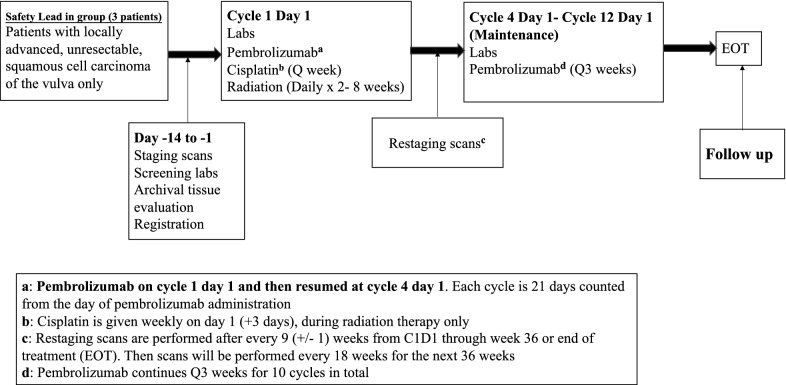
Treatment schema for the first three patients with unresectable disease as part of the safety cohort

**Fig. 2:**
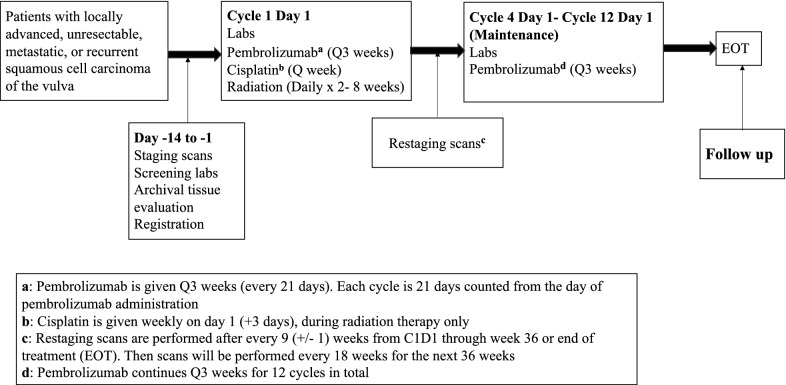
Treatment schema

The purpose of this study is to capitalize on the immunogenic properties of concurrent cisplatin/radiation via addition of pembrolizumab. Addition of pembrolizumab to cisplatin-sensitized radiation (Cis-RT) therapy is hypothesized to increase overall response rate, and recurrence free survival via increased cytotoxic T-cell engagement and activation. Based on this hypothesis, immunotherapy would need to be given concurrently with Cis-RT. Unlike CTLA-4 inhibitors, pembrolizumab has not been shown to have a dose-dependent relationship to toxicity with the currently utilized dose of 200 mg/m^2^ every 3 weeks. Due to this, a phase 1 trial with varying doses of pembrolizumab would not be instructive regarding toxicity and would likely impact potential efficacy. Also, due to the long half-life and frequency of administration, to test the hypothesis of synergy outlined in this study, pembrolizumab has to be present when Cis-RT is given. This makes a phase 2 trial design with pembrolizumab lead-in less ideal. In light of this, we decided to pursue a single-arm phase 2 clinical trial of concurrent Cis-RT and pembrolizumab. Finally, published and emerging toxicity data from immunotherapy plus chemoradiation combinations in other solid tumors have been reassuring, as described in the discussion section. The first three (3) patients with locally advanced or unresectable disease will receive cycle 1 of pembrolizumab followed by a break and resumption of pembrolizumab at cycle 4 and as part of a safety cohort. These patients will receive a total of 10 cycles of pembrolizumab (Fig. 1). All other patients, including the fourth patient with locally advanced/unresectable disease, will receive weekly cisplatin and pembrolizumab every 3 weeks, concurrently with daily radiation therapy (Fig. 2). Following completion of Cis-RT, patients will continue pembrolizumab maintenance for a total of 10 cycles in the safety cohort or 12 cycles for all other participants. Archived tissue will be used for MSI, PD-L1 and TIL stratification post hoc. Imaging will be performed at baseline and every 3 cycles (21-day cycles) as per standard-of-care. Laboratory analysis will occur on the first day of each cycle. We will collect and evaluate HPV status, PD-L1, and MSI status prior to therapy, but this will not be used for eligibility. Radiographic imaging will be performed every 9 weeks and overall response rate will be determined by RECIST 1.1 criteria.

### Patient characteristics

For this study, women ≥ 18 years with histologically or cytologically confirmed unresectable, incompletely resected, recurrent, or metastatic squamous cell carcinoma of the vulva will be eligible. Patients with unresectable disease defined as having T2 or T3 primary tumors (N0-3, M0) not amenable to surgical resection by standard radical vulvectomy will be eligible. Patients who have received prior chemotherapy, immunotherapy or radiation therapy will also be eligible. For patients who have received prior radiation therapy, re-irradiation to a previously treated site will not be permitted (Table 1).

**Table 1:** Inclusion and exclusion criteria

Inclusion criteria	Exclusion criteria
Histologically or cytologically confirmed unresectable, incompletely resected, recurrent, or metastatic squamous cell carcinoma of the vulva. Patients with unresectable disease are defined as T2 or T3 primary tumors (N0-3, M0) not amenable to surgical resection by standard radical vulvectomy	Patients who in the opinion of the investigator cannot safely receive a minimum of 30 Gy in 10 fractions are not eligible for the trial
Participants must have measurable disease based on RECIST 1.1. Lesions situated in a previously irradiated area are considered measurable if progression has been demonstrated in such lesions	Participants who have received prior systemic anti-cancer therapy including investigational agents within 4 weeks prior to first dose of study treatment
Documented Microsatellite stability status by routine methods including MMR IHC, MSI PCR or MSI by Next Generation Sequencing (NGS)	Participants who have received a live vaccine within 30 days prior to the first dose of study drug
Participants with no prior therapy are eligible and patients with recurrent disease must not have had more than two lines of cytotoxic therapy	Participants with a history of gastrointestinal or colovesicular fistulae
ECOG performance status of 0 or 1	Has active autoimmune disease that has required systemic treatment in the past 2 years (i.e. with use of disease modifying agents, corticosteroids or immunosuppressive drugs)
Absolute neutrophil count (ANC): ≥ 1500/µL Platelets: ≥ 100,000/µL Hemoglobin: ≥ 9.0 g/dL or ≥ 5.6 mmol/L Creatinine: ≤ 1.5 × ULN AST (SGOT) and ALT (SGPT): ≤ 2.5 × ULN Total bilirubin: ≤ 1.5 × ULN	Has known active CNS metastases and/or carcinomatous meningitis

*ULM* upper limit of normal

### Study procedures

After review and signing of written informed consent and trial registration, participants will be screened for eligibility and participation in the clinical trial. Pembrolizumab may be administered up to 1 day before or after the scheduled day 1 of each cycle due to administrative reasons. Cisplatin should be given on day 1 of every cycle. At some centers, cisplatin is administered on day 3 for administrative reasons and this will be allowable on study after notification of the PI during enrollment. The first 3 patients receiving definitive chemoradiation will receive pembrolizumab on cycle 1 only and then resume on cycle 4 after completion of chemoradiation (Fig. 1). This is for the first 3 patients with locally advanced, unresectable disease only. All other patients will be treated with concurrent pembrolizumab-cisplatin-radiation throughout (Fig. 2). For logistical reasons, patients are allowed to receive standard-of-care cisplatin-radiation locally. For these patients, radiation treatment plans are to be submitted to the radiation oncologist PI. These patients must also be available for AE assessments in person on day 1 of every cycle (pembrolizumab administration days) and weekly AE assessments by telephone. All trial treatments will be administered on an outpatient basis. Radiation therapy can be administered before or after immunotherapy or chemotherapy.

Pembrolizumab 200 mg will be administered as a 30-min IV infusion every 3 weeks. Pembrolizumab will be administered first, prior to administration of cisplatin. Pembrolizumab will be continued for a total of 12 cycles, except for patients in the safety cohort who will receive a total of 10 cycles. After premedication, cisplatin 40 mg/m^2^ will be administered as a 30–60 min IV infusion (depending on institutional guidelines) weekly during radiation therapy. Cisplatin should be administered at the beginning of the week during radiation therapy. To allow for variation of administration of cisplatin at various clinical sites, cisplatin administration up to day +3 will be allowed. This request will be noted prior to patient enrollment. Cisplatin will only be administered during radiation therapy, minimum of 2 weeks, maximum of 8 weeks.

For patients with locally advanced or unresectable disease, external beam radiotherapy will be directed to the vulva and regional lymph nodes as determined by primary tumor location, including but not limited to inguinofemoral, external and internal iliac nodal regions. Treatment will adhere as closely as possible to standard of care. Any deviations will be submitted for approval to the radiation oncology PI prior to initiation of therapy. Patients with localized recurrent and/or metastatic disease with vulvar or inguinal and/or pelvic involvement and no prior history of radiation therapy are also candidates for tumor-directed radiotherapy to the site of recurrence. Patients with recurrent metastatic disease and a history of prior radiation can be considered if the target lesions are outside the prior radiation field, symptomatic, or if in the opinion of the investigator the patient can safely receive and benefit from additional radiation. CT simulation is required to define the gross tumor volume (GTV), clinical target volume (CTV), and planning target volume (PTV). The CT scan must be acquired in the same position and immobilization device as for treatment. The vulvar GTV and the groin GTV when inguinal nodes are unresectable will receive 68–70 Gy in 32–38 fractions of 1.8–2.2 Gy per fraction, provided organs at risk (OAR) metrics are met. Dose painting is permitted. Treatment will be delivered once daily, 5 days per week for the treatment duration. Breaks from treatment should be minimized and reasons for breaks must be documented. The groin PTV when high-risk positive nodal features are present after lymph-node dissection will receive 60 Gy in 30 fractions. Uninvolved pelvic or inguinal nodes should receive 45–50.4 Gy. Patients with unresectable/unresected pelvic nodes will receive radiation to a dose of 64 Gy, as OARs allow. For patients with a prior history of radiation, no overlap of previously treated sites will be permitted. Thoracic metastasis will not to be irradiated. Treatment of bone metastases are allowed. Patients who in the opinion of the investigator cannot safely receive a minimum of 30 Gy in 10 fractions for metastatic disease are not eligible for the trial.

### Expected toxicities and management

Toxicity will be graded using CTCAE version 5.0, and treatment criteria will be evaluated prior to each cycle (Table 2). Participants will be followed for up to 3 years or until death, whichever occurs first. Participants removed from protocol therapy for unacceptable adverse event(s) will be followed until resolution or stabilization of the adverse event. Survival status will be checked every 6 months during that time. Participants removed from study for unacceptable adverse events will be followed until resolution or stabilization of the adverse event. Expected toxicities from cisplatin include nausea, diarrhea, nephrotoxicity, tinnitus, ototoxicity, neutropenia, and thrombocytopenia. In the cases of grade 3 or 4 nausea, cisplatin would be held, and dose reduced (Table 3). Commonly seen adverse events seen with pembrolizumab include immune-mediated pneumonitis, colitis, hepatitis, hypophysitis, hypo/hyperthyroidism, thyroiditis, type 1 diabetes mellitus, nephritis and rashes including; Stephens-Jonson syndrome (SJS) and Toxic epidermal necrolysis (TEN). Similarly, management outlines for neutropenia and thrombocytopenia are also shown in Table 3. In the event of suspected or confirmed immune-related adverse events (irAEs) attributed to pembrolizumab, guidelines for management are detailed in Table 4. Adverse events from radiation therapy include rashes, wounds, and other cutaneous manifestations, dysuria, and diarrhea.

**Table 2:** Treatment criteria for the first cycle and subsequent cycles

Cycle 1 criteria	Subsequent cycles
Absolute neutrophil count ≥ 1500/mcL Platelets ≥ 100,000/mcL Hemoglobin ≥ 9 g/dL Total bilirubin ≤ 1.5 × institutional upper limit of normal AST (SGOT)/ALT (SGPT) ≤ 2.5 × institutional upper limit of normal Creatinine ≤ 1.5 × ULN or creatinine clearance ≥ 50 mL/min for subjects with creatinine levels above institutional ULN All toxicities of previous therapy (aside from alopecia) must have resolved to ≤ grade 1 ECOG performance of 0 or 1 No evidence of life-threatening medical problems	Absolute neutrophil count ≥ 500/mcL Platelets ≥ 50,000/mcL AST (SGOT)/ALT (SGPT) ≤ 2.5 × institutional upper limit of normal Creatinine ≤ 1.5 × ULN or creatinine clearance ≥ 50 mL/min for subjects with creatinine levels above institutional ULN All toxicities of previous cycles must have resolved to ≤ grade 2 ECOG performance of 0 to 2 No evidence of life-threatening medical problems

*ULM* upper limit of normal

**Table 3:** Toxicity and treatment holds

	Management/next dose for cisplatin	Management/next dose for pembrolizumab
Nausea/vomiting		
≤ Grade 1	No change in dose	No change in dose
Grade 2	No change in dose	No change in dose
Grade 3	Hold^a^ until < Grade 2. Resume at one dose level lower, if indicated.	No change in dose^b,c^
Grade 4	Hold^a^ until < Grade 2. Resume at two dose levels lower.	No change in dose^b,c^
Diarrhea		
≤ Grade 1	No change in dose	No change in dose
Grade 2	No change in dose	No change in dose
Grade 3	Hold^a^ until < Grade 2. Resume at one dose level lower, if indicated	No change in dose^b,c^
Grade 4	Hold^a^ until < Grade 2. Resume at two dose levels lower	No change in dose^b,c^
Neutropenia		
≤ Grade 1	No change in dose	No change in dose
Grade 2	No change in dose	No change in dose
Grade 3	No change in dose	No change in dose
Grade 4	Hold^d^ until < Grade 3. Resume at current dose.	Hold until ≤ Grade 2
Thrombocytopenia		
≤ Grade 1	No change in dose	No change in dose
Grade 2	No change in dose	No change in dose
Grade 3	Hold^d^ until < Grade 2. Resume at same dose level	No change in dose
Grade 4	Hold^d^ until < Grade 2. Resume at same dose level	Hold until ≤ Grade 2

^a^Participants requiring a delay of > 2 weeks should go off protocol therapy

^b^Please see pembrolizumab adverse event management in Table 4

^c^As long as AE is unrelated to Pembrolizumab

^d^Participants requiring a delay of > 4 weeks should go off protocol therapy

**Table 4:** Pembrolizumab toxicity and management

Immune related Adverse Events (irAEs)	Toxicity grade (CTCAE V5.0)	Action with pembrolizumab	Corticosteroid and/or other therapies	Monitoring and follow-up
Pneumonitis	Grade 2	Withhold	Administer corticosteroids (initial dose of 1–2 mg/kg prednisone or equivalent) followed by taper Add prophylactic antibiotics for opportunistic infections	Monitor participants for signs and symptoms of pneumonitis Evaluate participants with suspected pneumonitis with radiographic imaging and initiate corticosteroid treatment
	Grade 3 or 4, or recurrent Grade 2	Permanently discontinue		
Diarrhea/Colitis	Grade 2 or 3	Withhold	Administer corticosteroids (initial dose of 1–2 mg/kg prednisone or equivalent) followed by taper	Monitor participants for signs and symptoms of enterocolitis (i.e., diarrhea, abdominal pain, blood or mucus in stool with or without fever) and of bowel perforation (i.e., peritoneal signs and ileus) Participants with ≥ Grade 2 diarrhea suspecting colitis should consider GI consultation and performing endoscopy to rule out colitis Participants with diarrhea/colitis should be advised to drink liberal quantities of clear fluids. If sufficient oral fluid intake is not feasible, fluid and electrolytes should be substituted via IV infusion
	Grade 4 or recurrent Grade 3	Permanently discontinue		
AST or ALT elevation or Increased Bilirubin	Grade 2^a^	Withhold	Administer corticosteroids (initial dose of 0.5–1 mg/kg prednisone or equivalent) followed by taper	Monitor with liver function tests (consider weekly or more frequently until liver enzyme value returned to baseline or is stable)
	Grade 3^b^ or 4^c^	Permanently discontinue	Administer corticosteroids (initial dose of 1–2 mg/kg prednisone or equivalent) followed by taper	
Type 1 diabetes mellitus (T1DM) or Hyperglycemia	New onset T1DM or Grade 3 or 4 hyperglycemia associated with evidence of β–cell failure	Withhold^d^	Initiate insulin replacement therapy for participants with T1DM Administer anti-hyperglycemic in participants with hyperglycemia	Monitor participants for hyperglycemia or other signs and symptoms of diabetes
Hypophysitis	Grade 2	Withhold	Administer corticosteroids and initiate hormonal replacements as clinically indicated	Monitor for signs and symptoms of hypophysitis (including hypopituitarism and adrenal insufficiency)
	Grade 3 or 4	Withhold or permanently discontinue^d^		
Hyperthyroidism	Grade 2	Continue	Treat with non-selective beta-blockers (e.g., propranolol) or thionamides as appropriate	Monitor for signs and symptoms of thyroid disorders
	Grade 3 or 4	Withhold or permanently discontinue^d^		
Hypothyroidism	Grade 2, 3, or 4	Continue	Initiate thyroid replacement hormones (e.g., levothyroxine or liothyronine) per standard of care	Monitor for signs and symptoms of thyroid disorders
Nephritis and renal dysfunction: grading according to increased creatinine or acute kidney injury	Grade 2	Withhold	Administer corticosteroids (prednisone 1–2 mg/kg or equivalent) followed by taper	Monitor changes of renal function
	Grade 3 or 4	Permanently discontinue		
Myocarditis	Grade 1 or 2	Withhold	Based on severity of AE administer corticosteroids	Ensure adequate evaluation to confirm etiology and/or exclude other causes
	Grade 3 or 4	Permanently discontinue		
All Other immune-related AEs	Intolerable/persistent Grade 2	Withhold	Based on severity of AE administer corticosteroids	Ensure adequate evaluation to confirm etiology or exclude other causes
	Grade 3	Withhold or discontinue based on the event^e^		
	Grade 4 or recurrent Grade 3	Permanently discontinue		

^a^ AST/ALT: > 3.0–5.0 × ULN if baseline normal; > 3.0–5.0 × baseline, if baseline abnormal; bilirubin: > 1.5–3.0 × ULN if baseline normal; > 1.5–3.0 × baseline if baseline abnormal

^b^ AST/ALT: > 5.0 to 20.0 × ULN, if baseline normal; > 5.0–20.0 × baseline, if baseline abnormal; bilirubin: > 3.0–10.0 × ULN if baseline normal; > 3.0 × 10.0 × baseline if baseline abnormal

^c^ AST/ALT: > 20.0 × ULN, if baseline normal; > 20.0 × baseline, if baseline abnormal; bilirubin: > 10.0 × ULN if baseline normal; > 10.0× baseline if baseline abnormal

^d^ The decision to withhold or permanently discontinue pembrolizumab is at the discretion of the investigator or treating physician. For participants with Grade 3 or 4 immune-related endocrinopathy where withhold of pembrolizumab is required, pembrolizumab may be resumed when AE resolves to ≤ Grade 2 and is controlled with hormonal replacement therapy or achieved metabolic control (in case of T1DM)

^e^ Events that require discontinuation include but are not limited to: Guillain–Barre Syndrome, encephalitis, Stevens-Johnson Syndrome and toxic epidermal necrolysis

Severe diarrhea could be from cisplatin, radiation, or pembrolizumab, and precise attribution might be challenging in some cases. To prevent patients receiving definitive therapy from unnecessary treatment breaks and delays due to suspected superimposed immune-related colitis, the first three patients with locally advanced/unresectable disease will get pembrolizumab on cycle 1 day 1 and omit further pembrolizumab until cycle 4 day 1. If no unexpected toxicities are seen, all remaining patients in this category will receive pembrolizumab uninterrupted every 3 weeks.

### Translational studies

Multiplex IHC will be performed on all archival samples for determination of; (i) CD3+, CD8+TILs, CD8+/CD4+FOXP3+TIL ratio, CD137+CD8+TILs, CD137+CD8+/CD4+FOXP3+TIL ratio, peritumoral lymphocytes and correlation with response; (ii) expression of immune checkpoints including TIM-3, LAG-3, CTLA-4, PD-L2, PD-L1, PD-1, and correlation with response; (iii) targeted next generation T-cell receptor sequencing (Adaptive biotechnologies) to determine T-cell clonality and correlate with response. Multiparameter flow cytometry will be used to determine the effect of treatment on circulating CD8+, CD4+FOXP3+T-cells. Cytokine analysis will also be performed to measure IFN-α, IFN-β, TNF-α, IL-6, IL-10, IL-12, and IL-2 levels and correlated with response. This will allow for comprehensive baseline profiling that will be correlated with treatment response. This approach has been validated as a means to determine the potential predictive value of immune cell phenotype and spatial distribution relative to the tumor [28]. This might allow for a better understanding of patients who might benefit from this form of combined therapy. We will collect HPV status, and MSI status at baseline. Identification of microsatellite instability resulting from defects in the mismatch repair pathway and specifically within MLH1, MSH2, MSH6 and PMS2 have been suggested to portend a significantly improved response to checkpoint blockade [29]. This information will be correlated with treatment response. One of the key mechanisms via which cisplatin-sensitized radiation therapy mediates its immunogenic effect is via calreticulin exposure, release of ATP, induction of programmed death receptor 1-ligand (PD-L1) and high-mobility protein box-1 (HMGB-1) [20, 21]. As such, we will measure baseline serum HMGB1 and at cycle 1, day 1 (D1), D5, D8, D12, D15, D19, D22 and D26 while on combination therapy via commercially available ELISA assays.

### Statistical considerations

The target enrolment is 24 patients. The primary endpoint for this study is overall response rate (ORR). This sample size calculation is based on an ORR of ≥ 60%. For recurrent metastatic disease, there are is no standard of care. The ORR estimation is informed by data showing an ORR of 40% in patients with advanced or recurrent metastatic vulvar cancer treated with platinum-based combination therapy [4]. Single-agent chemotherapy has an ORR of about 12% [5]. Patients with primary disease who are not candidates for upfront surgery have a reported response rates from 55% [30] to 64% [11]. Only patients who undergo treatment on protocol will be eligible for analysis. Patients who sign consent and do not undergo any treatment will be ineligible for evaluation. With n = 24, the power to reject Ho: ORR ≤ 30% in favor of H1: ORR ≥ 60% will be 89% at a target significance level of 0.05 for a one-sided exact Binomial test (12 or more responses are required to reject Ho in favor of H1). Six-month Recurrence Free Survival (RFS-6) is the secondary endpoint. Exploratory biomarkers include; Predictive values of baseline dMMR/MSI-status and PD-L1; Anti-tumor inflammatory responses of cGas-STING- pathway, systemic inflammatory cytokines, and circulating T-cell receptor repertoire.

### Analysis strategy

Exact Binomial test and 95% CI for ORR (primary endpoint). For the secondary objectives of RFS-6, a point estimate with exact 95% CI and Kaplan–Meier estimate will be used for the RFS distribution. Operating characteristics of prediction (sensitivity, specificity, positive- and negative predictive values with 95% CI) for the baseline dMMR/MSI-status and PD-L1, and descriptive analysis will be used for anti-tumor inflammatory cytokines and HMGB-1 levels.

## Discussion

There are numerous clinical trials that support safety of the combination of chemotherapy with immune checkpoint blockade [31–33]. In addition, there is prospective safety data for combining chemoradiation with immune checkpoint blockade. Phase 2 and 3 clinical trials in NSCLC evaluating concurrent platinum-based chemotherapy with RT and PD-L1 blockade reported an incidence of about 30% in adverse related events [27, 34]. This is not significantly higher than what would be expected for chemoradiation treatment alone. A phase 1 trial by Tang et al. evaluated the combination of hypofractionated radiotherapy; 50 Gy in 4 fractions or 60 Gy in 10 fractions, with ipilimumab in patients with metastatic solid tumor malignancies [35]. In this study, 34% of patients developed grade 3 toxicity with colitis being the most common adverse event. Of note, 23% of the patients treated on this trial derived benefit via abscopal effects [35]. Another phase I trial combined pembrolizumab with various courses of radiation therapy; 30 Gy in 3 fractions for osseous metastasis, 50 Gy in 5 fractions for central lung tumors and 45 Gy in 3 fractions for other sites [36]. Only 6% of the 62 participants in this trial experienced grade 3 toxicities. The authors also reported abscopal effects in 13.2% of these patients [36]. NRG-GY017 is an ongoing phase I clinical trial evaluating an anti-PD-L1 inhibitor, atezolizumab, in patients with node positive Stage IB2, II, IIIB, or IVA cervical cancer. Similar to this trial, both arms of the study include the administration of immune checkpoint blockade concurrently with cisplatin-sensitized radiation therapy at doses similarly used for GYN malignancies, and there have been no adverse safety signals reported (NCT 03738228). As a safety precaution, the first three patients with locally advanced unresectable disease treated on this trial with receive pembrolizumab on cycle 1 and then resume on cycle 4 and on (Fig. 1).

Several preclinical studies show that combination cisplatin and radiation therapy increases the immunogenetic effect of each treatment modality in ways that can be further enhanced by PD-1/PD-L1 blockade. By adding pembrolizumab early during the delivery of Cis-RT, the opportunity for antigen capture and presentation, DC priming and T-cell activation is potentially increased. The pragmatic design of this trial also allows for collection of translational correlatives to evaluate if a systemic anti-tumor inflammatory response is being generated by this triplet combination.

In a rare disease with limited options in the recurrent setting, addition of immune checkpoint blockade to cisplatin-sensitized radiation could provide durable disease control by engaging the immune system.

## Data Availability

At the completion of the trial, de-identified data will be available upon request.

## Abbreviations

PD-L1: Programmed death receptor 1-ligand
PD-L2: Programmed death receptor 2-ligand
HPV: Human papillomavirus
TIL: Tumor infiltrating Lymphocytes
MSI: Microsatellite instability
dMMR: Deficient mismatch repair
IL-2: Interleukin 2
DC: Dendritic cell
M6P: Mannose-6-phosphate
RT: Radiation therapy
PFS: Progression-free survival
PI: Principal investigator
AE: Adverse event
OS: Overall survival
NSCLC: Non small cell lung cancer
IFN-γ: Interferon gamma
HMGB-1: High-mobility protein box-1
MHC: Major histocompatibility complex
AE: Adverse events
GTV: Gross tumor volume
CTV: Clinical tumor volume
PTV: Planning tumor volume
OAR: Organs at risk
CTCAE: Common terminology criteria for adverse events
IHC: Immunohistochemistry
ELISA: Enzyme-linked immunosorbent assay
ORR: Overall response rate
RFS: Relapse free survival
